# Thoracic Splenosis in the Setting of Abdominal Trauma

**DOI:** 10.7759/cureus.27851

**Published:** 2022-08-10

**Authors:** Olufunmilola Ajala, Linus Yoe, Tess Decatur, Owen Cole

**Affiliations:** 1 Internal Medicine, The Brooklyn Hospital Center, Brooklyn, USA

**Keywords:** splenosis, splenectomy, abdominal trauma, splenosis, hypoxia

## Abstract

The uncommon case of thoracic splenosis is presented in this paper. A patient presents to the hospital with the complaint of dyspnea on exertion. He is incidentally found to have thoracic splenosis. The case of thoracic splenosis is a vital topic to discuss in order to accurately diagnose, recognize, treat symptoms, and explore how it can exacerbate pulmonary or cardiology pathology.

## Introduction

Splenosis is an uncommon complication of splenic and diaphragmatic injury or elective splenectomy [1]. Splenosis is usually an incidental finding on imaging. It is defined as the autotransplantation of ectopic splenic tissue to abnormal sites of the body [2] following abdominal trauma or surgery. Splenic tissue can gain access to the left pleural space through a diaphragmatic tear or a congenital defect, and parasitizes blood supply from the pleura, chest wall, or diaphragm [3]. Splenosis can be seen on CT scans and confirmed with a nuclear medicine sulfur colloid liver scan [4]. This study is performed using a gamma camera with low energy collimator following the IV administration of 4.2 mCi of Tc 99m sulfur colloid into the left hand [4-5]. Patients can be symptomatic or asymptomatic [6]. Past case studies have attributed symptoms such as hemoptysis, cough, and pleuritic chest pain to thoracic splenosis [7-9]. However, patients with these symptoms also presented with other comorbidities, such as pneumonia or questionable malignancy, in addition to thoracic splenosis [7-9].

The case of thoracic splenosis is an important topic because there is limited research regarding the most accurate diagnosing mode. Medical providers need to be able to diagnose it so that unnecessary procedures can be avoided. Limited studies are available regarding whether splenosis can contribute to or exacerbate cardiac and pulmonary symptoms.

## Case presentation

A 58-year-old African American male presented to the ED with shortness of breath on exertion associated with intermittent dry cough and dizziness for one week. He was admitted to the hospital for further evaluation of presenting symptoms. The patient’s past medical history included a gunshot wound to the abdomen status post exploratory laparotomy and splenectomy (in 1988), hypertension, cor pulmonale, obstructive sleep apnea, and chronic anemia. He denied headache, fever, chills, runny nose, sore throat, myalgias, chest pain, orthopnea, abdominal pain, and changes in urinary or bowel habits. 

The patient was tachycardic and afebrile. Physical examination revealed an obese middle-aged man. Breath sounds were clear on auscultation bilaterally. His oxygen saturation was 83%, and he was placed on 3 liters of oxygen via a nasal cannula which increased his O2 saturation to 95%. There was no leukocytosis, and electrolyte values were within normal limits. Mild anemia was present (hemoglobin 12). He was negative for influenza, mycoplasma, legionella, or chlamydia and his COVID-19 PCR test was negative. Arterial blood gas revealed hypercapnia and hypoxemia (pH 7.38, PCO2 64 mmHg, PO2 45 mmHg, bicarbonate 38 mEq/L, O2 80). Inflammatory markers were within normal limits.

Differential diagnoses for the clinical presentation included decompensated congestive heart failure, cor pulmonale, lung malignancy (including carcinoma, metastasis, and other benign growths), pulmonary embolism, interstitial lung disease, obstructive sleep apnea (OSA), and obesity hypoventilation syndrome (OHS).

The patient’s transesophageal echocardiogram showed moderate concentric left ventricular hypertrophy (LVH), moderate tricuspid regurgitation, moderate pulmonary hypertension, moderate right ventricular dilation, and moderately reduced right ventricular systolic function.

**Figure 1:**
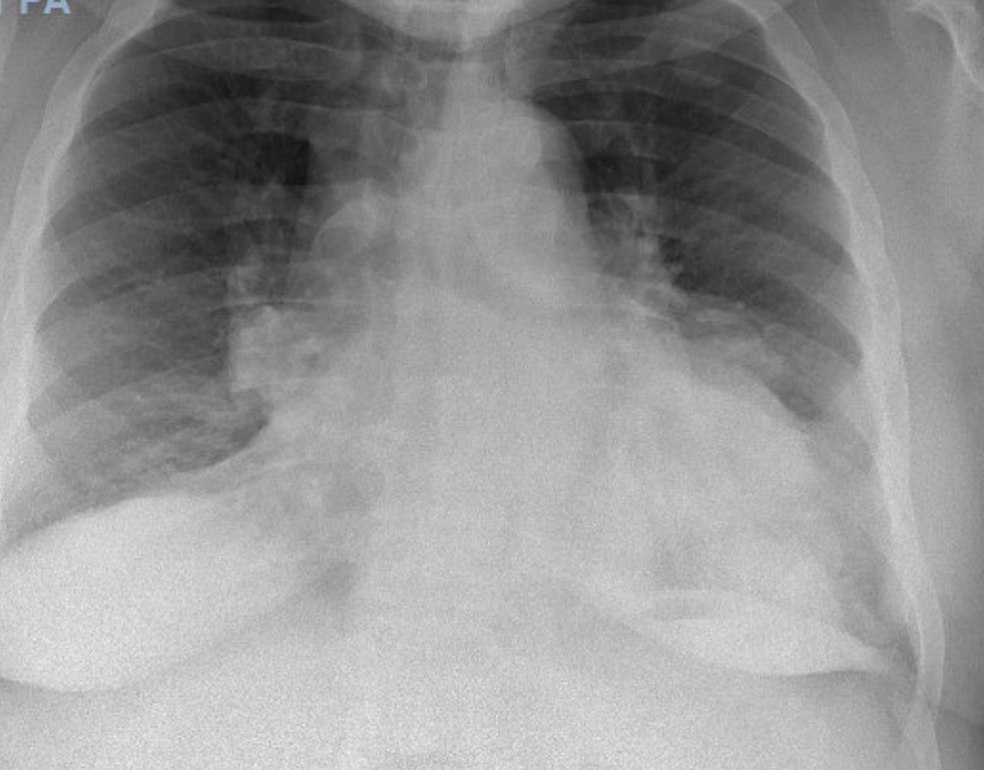
Chest X-ray. Patchy opacities in the left lung base and mild prominence of the bilateral hilar nodes.

**Figure 2:**
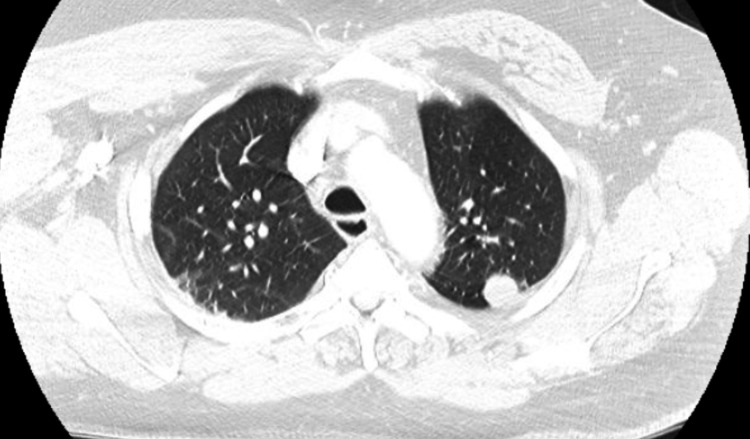
CT angiography chest. Multiple scattered pleural-based nodules and masses in the left hemithorax abutting the left lateral aspect of the mediastinum and along the diaphragm, which were concerning for a neoplastic etiology.

**Figure 3:**
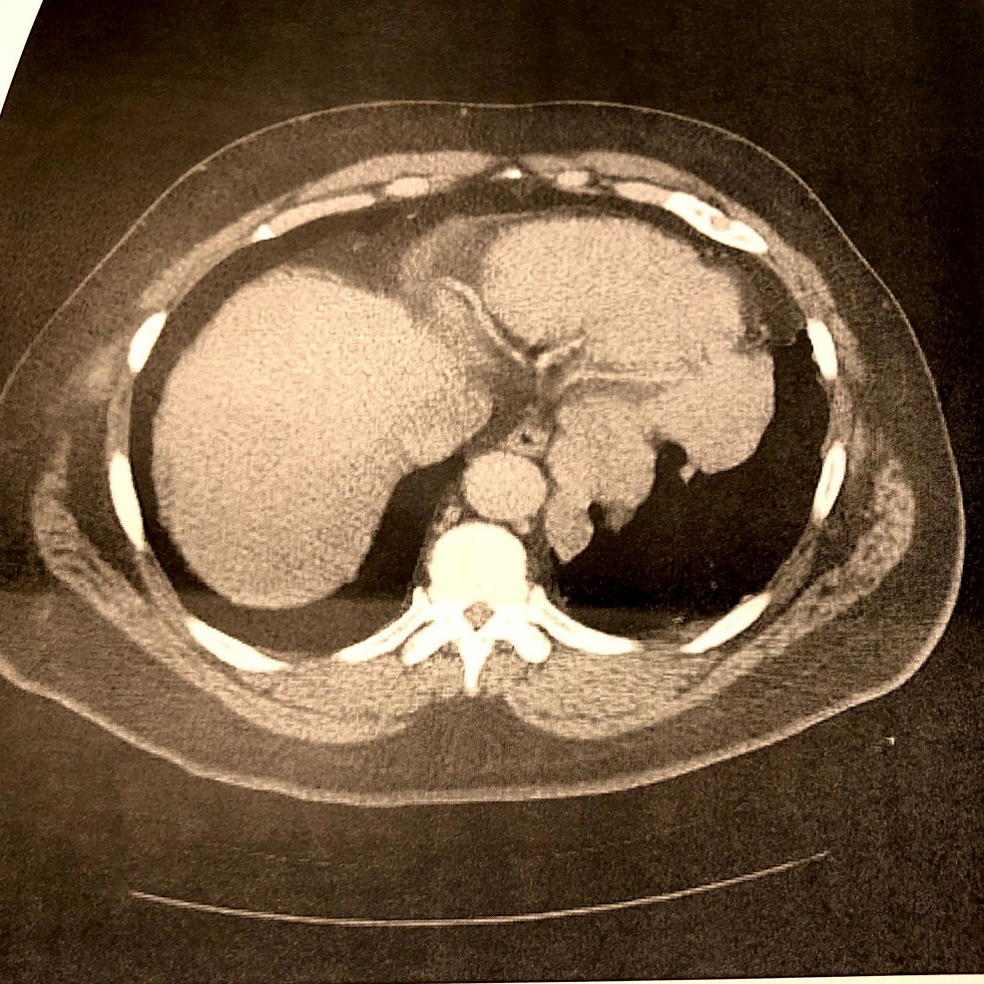
CT abdomen and pelvis w/contrast. Multiple left lower masses are seen.

**Figure 4:**
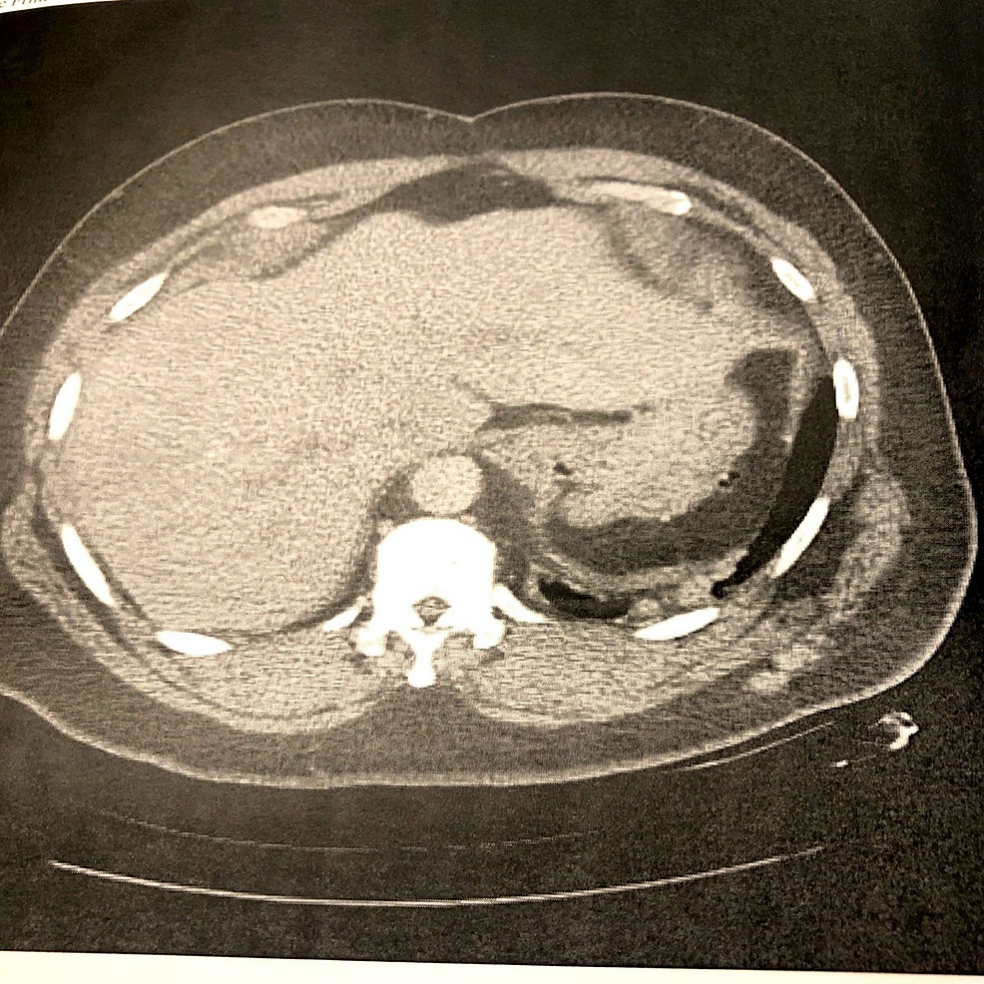
CT abdomen/pelvis w/ contrast.

**Figure 5:**
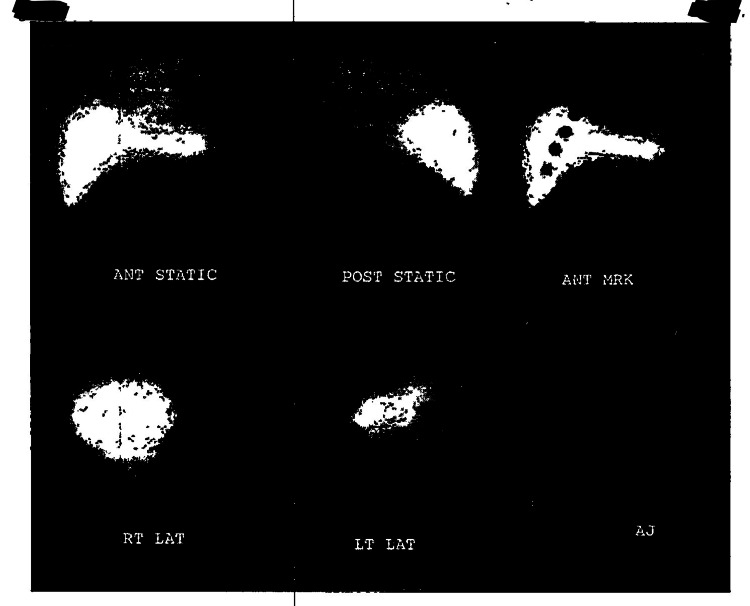
The nuclear medicine sulfur colloid liver spleen scan. The nuclear medicine sulfur colloid liver spleen scan confirmed that the nodules consisted of splenic tissue.

The patient's chest X-ray showed patchy opacities in the left lung base and mild prominence of the bilateral hilar nodes (Figure 1), which was similar to a chest X-ray in 2018. The CT angiography of the chest revealed multiple, scattered pleural-based nodules and masses in the left hemithorax abutting the left lateral aspect of the mediastinum and along the diaphragm, which were concerning for a neoplastic etiology (Figure 2). There was no evidence of pulmonary embolism. CT abdomen and pelvis were completed to further evaluate the findings on the CT angiography of the chest. CT abdomen and pelvis with contrast revealed masses and nodules in the left lower lobe and diaphragm were compatible with splenic tissue. A nuclear medicine sulfur colloid liver spleen scan was completed and confirmed that the nodules consisted of splenic tissue. The study's findings, in this case, revealed a well-outlined heart and abdominal aorta. The liver was normal in size, morphology and demonstrated homogenous uptake. There was no uptake in the spleen. There were nodular and band-like areas of abnormal uptake in the left hemithorax, particularly along the left heart border and at the base of the left lung. The findings of absent uptake in the spleen and several foci of abnormal uptake in the left hemithorax, evident at the left lung base and along the left heart border, were consistent with splenosis. Figures 3-4 reveal the splenosis on CT and Figure 5 shows the uptake of Tc 99m in the nuclear medicine sulfur colloid liver spleen scan.

The patient was treated for symptoms which included Duonebs and oxygen supplementation as needed. His symptoms improved. He was discharged with continuous positive airway pressure (CPAP) at night and advised to follow up in a pulmonology and cardiology clinic.

## Discussion

This case of thoracic splenosis is an interesting case because it can be mistaken for lung nodules and malignancy. The patient, in this case, had comorbidities, including obesity, cor pulmonale, OHS/OSA, pulmonary hypertension, and LVH, which likely contributed to his dyspnea. However, the thoracic splenosis may have also contributed to his symptoms. Compared to other published case reports, there is limited information regarding whether or not thoracic splenosis can exacerbate the severity of cardiac and pulmonary pathologies such as heart failure, pulmonary hypertension, and OSA/OHS, as seen in this patient. Complications of splenosis can include small bowel obstruction, shortness of breath, pleuritic chest pain, hemoptysis, and respiratory failure [1,2,3,4]. However, this patient's symptoms were likely due to his other comorbidities, and the thoracic splenosis was an incidental finding.

The frequency of splenosis varies but typically ranges from 26 to 65% following trauma and 16 to 20% following elective splenectomy [1,2,3,4]. Thoracic splenosis occurs in approximately 18% of patients with combined diaphragmatic and splenic injuries and can be seen after penetrating injuries [5]. Compared with other case reports, the timeframe of appearance of splenosis is usually 20 years after the time of abdominal trauma [6], which is similar to the timeframe of appearance in this patient. Historically, diagnosis relied on invasive procedures and surgery for biopsy of the ectopic splenic tissue [1]. However, with appropriate clinical judgment and proper nuclear medicine scans, a diagnosis can be made without needing biopsy, surgery, or invasive procedures. Similar to other case reports, treatment is primarily supportive and includes symptom control. Splenosis is usually benign, so invasive surgical procedures for removing splenosis are usually not performed [7]. Risks, such as bleeding and surrounding organ damage, outweigh the benefits of surgical removal of splenosis [8]. Prior case reports discussed how CT-guided biopsies, thoracotomies, and needle aspirations have been used in the past to evaluate/confirm lung nodules consistent with splenosis [8]. In this case report, non-invasive imaging was used to confirm the diagnosis.

In this case, the patient was treated for symptoms with improved clinical status. The question remains whether thoracic splenosis can contribute to symptoms caused by OSA, obstructive pulmonary disease, and heart failure. The theory of splenosis providing a protective factor/slight immunity in those without spleens has been proposed in past case reports [9]. However, patients who have undergone splenectomies are still advised to stay up to date with immunizations, especially against encapsulated organisms, regardless of whether or not splenosis is present because immune functions of the spleen are reduced [9]. In this case report, the patient was not immunocompromised and was up to date with his vaccinations, so it is unknown whether the splenosis provided a protective factor for him.

## Conclusions

In this case, the patient was treated for symptoms with improvement in clinical status. He did not undergo any invasive procedures. The question remains whether thoracic splenosis can be a protective factor or contribute to exacerbating medical conditions such as OSA, obstructive pulmonary disease, heart failure, and other cardiac and pulmonary pathologies. The theory of splenosis providing a protective factor/slight immunity in those without spleens has been proposed. However, patients who have undergone splenectomies are still advised to stay up to date with immunizations, especially against encapsulated organisms, regardless of whether or not splenosis is present because immune functions of the spleen are reduced.
